# Non-antibiotic strategies for prevention and treatment of internalized *Staphylococcus aureus*

**DOI:** 10.3389/fmicb.2022.974984

**Published:** 2022-08-31

**Authors:** Jiangbi Li, Qiangqiang Wen, Feng Gu, Lijuan An, Tiecheng Yu

**Affiliations:** ^1^Department of Orthopedics, The First Hospital of Jilin University, Changchun, China; ^2^Department of Orthopedics, The Affiliated Hospital of Northwest University, Xi’an, China; ^3^Department of Rehabilitation Medicine, The Second Affiliated Hospital of Kunming Medical University, Kunming, China

**Keywords:** *Staphylococcus aureus*, bacterial persistence, internalization, nanoparticles, cell-penetrating peptides

## Abstract

*Staphylococcus aureus* (*S. aureus*) infections are often difficult to cure completely. One of the main reasons for this difficulty is that *S. aureus* can be internalized into cells after infecting tissue. Because conventional antibiotics and immune cells have difficulty entering cells, the bacteria can survive long enough to cause recurrent infections, which poses a serious burden in healthcare settings because repeated infections drastically increase treatment costs. Therefore, preventing and treating *S. aureus* internalization is becoming a research hotspot. *S. aureus* internalization can essentially be divided into three phases: (1) *S. aureus* binds to the extracellular matrix (ECM), (2) fibronectin (Fn) receptors mediate *S. aureus* internalization into cells, and (3) intracellular *S. aureus* and persistence into cells. Different phases require different treatments. Many studies have reported on different treatments at different phases of bacterial infection. In the first and second phases, the latest research results show that the cell wall-anchored protein vaccine and some microbial agents can inhibit the adhesion of *S. aureus* to host cells. In the third phase, nanoparticles, photochemical internalization (PCI), cell-penetrating peptides (CPPs), antimicrobial peptides (AMPs), and bacteriophage therapy can effectively eliminate bacteria from cells. In this paper, the recent progress in the infection process and the prevention and treatment of *S. aureus* internalization is summarized by reviewing a large number of studies.

## Introduction

*S. aureus* is a significant human pathogen that causes a variety of clinical illnesses. It is a prominent cause of bacteremia, infective endocarditis, osteoarthritis, skin and soft tissue infections, pleuropulmonary infections, and device-related infections (Tong et al., 2015). *S. aureus* infection has a significant socioeconomic impact in both developed and poor countries (Balasubramanian et al., 2017). For instance, a thorough analysis of skin and soft tissue infections between 2001 and 2009 revealed that hospitalized patients’ treatment expenditures in the United States ranged from $12,000 to $23,000 (Suaya et al., 2014). Sometimes, it is difficult to cure these infections completely, especially when they become chronic. The internalization of *S. aureus* by cells and bacterial biofilms is the major cause of persistent and difficult-to-treat infections (Richter et al., 2016). Recently, some studies have summarized the formation of and treatment strategies for biofilms in detail (Bhattacharya et al., 2015; Moormeier and Bayles, 2017; Suresh et al., 2019), and this paper will not elaborate any further on that topic.

**FIGURE 1:**
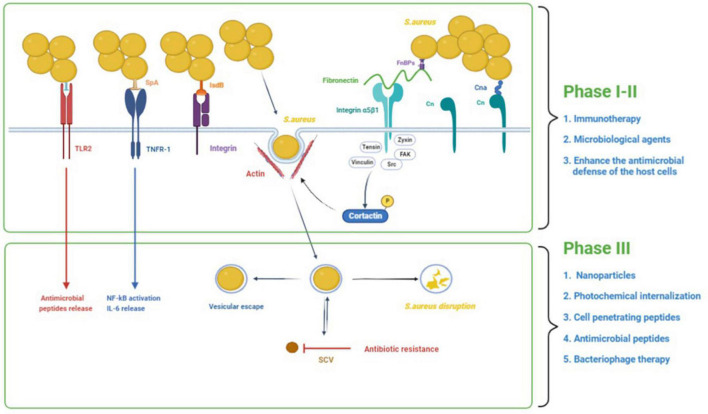
The process of *S. aureus* internalization into cells and the potential treatments. The process of *S. aureus* internalization can essentially be divided into three phases: (1) *S. aureus* binds to the extracellular matrix (ECM), (2) fibronectin (Fn) receptors mediate *S. aureus* internalization into cells, and (3) intracellular *S. aureus* and persistence into osteoblasts. In addition to binding to the ECM, *S. aureus* can also directly stimulate osteoblasts through its PAMPs, resulting in a variety of cellular reactions. In the first and second phases, immunotherapy and some microbial agents can effectively interfere with bacterial adhesion. Besides, some agents could enhance the antimicrobial defense of the host cells by inducing the production of antimicrobial peptides and decreasing the secretion of inflammatory cytokines. In the third phase, some treatments such as nanoparticles, photochemical internalization, cell-penetrating peptides, antimicrobial peptides, and bacteriophage therapy have significant bactericidal effects against intracellular *S. aureus*.

There is growing evidence that *S. aureus* has the ability to invade and persist within eukaryotic cells. *S. aureus* has been found in many non-phagocytic cells, such as human osteoblast cell lines, normal chick osteoblasts, mouse fibroblasts, mouse renal cells, bovine mammary epithelial cells, and human bronchial epithelial cells (Alexander and Hudson, 2001; Richter et al., 2016). *S. aureus* intracellularity has been proposed as an immune-evasive strategy to avoid identification by professional phagocytes (Fraunholz and Sinha, 2012). One of the most challenging aspects of treating intracellular *S. aureus* infections is getting enough antibacterial medicines to the intracellular bacteria. Many antibiotics have limited cell membrane penetration (β-lactams and aminoglycosides), intracellular unabiding retention (fluoroquinolones and macrolides), insufficient intracellular distribution, and low intracellular concentration (Zhou et al., 2018). Therefore, there is an urgent demand to develop novel therapies to treat intracellular *S. aureus* infections. The process of *S. aureus* internalization can essentially be divided into three phases (Figure 1): (i) *S. aureus* binds to the extracellular matrix (ECM), (ii) fibronectin (Fn) receptors mediate *S. aureus* internalization into cells, and (iii) intracellular *S. aureus* and persistence into cells (Wen et al., 2020). We will use osteoblasts as an example to illustrate the process of *S. aureus* internalization.

## The process of osteoblastic infection by *Staphylococcus aureus*

### *Staphylococcus aureus* binding to the bone extracellular matrix

*S. aureus*’*s* ability to infect bone, and more especially the osteoblast, is closely tied to its ability to bind the bone extracellular matrix (BEM) components (Heilmann, 2011). All of the proteins and glycans in the BEM are possible *S. aureus* binding sites. The extracellular matrix can foster the accumulation of *S. aureus* near osteoblasts. I collagen (Cn), bone sialoprotein, osteopontin, and fibronectin are the most studied because they interact directly with *S. aureus* (Josse et al., 2015). *S. aureus* binds to the BEM through its cell wall-anchored (CWA) proteins while contacting bone tissue in preparation for subsequent internalization. Cn represents approximately 90–95% of the organic fraction of the BEM, and the Cn adhesin (Cna) protein on the surface of *S. aureus* plays a function by adhering to Cn in the process of *S. aureus* infection in bone tissue (Patti et al., 1994). The N-terminal A domain (containing of N1, N2, and N3), the B repetitive sequence, the cell wall anchoring domain, and a brief cytoplasmic region comprise Cna proteins (Zong et al., 2005; Herman-Bausier et al., 2016). Cna primarily binds to Cn *via* a tightly wrapped mechanism (the “collagen hug” mechanism) (Herman-Bausier et al., 2016). The N1 and N2 domains of the N-terminal A domain (which has an IgG fold-like structure) and the B repetitive sequence are now known to be involved in this mechanism. *S. aureus* proteinaceous surface adhesins can be covalently attached to the cell wall peptidoglycan or surface-associated *via* various mechanisms such as ionic or hydrophobic interactions (Heilmann, 2011).

### Fibronectin receptors mediate *Staphylococcus aureus* internalization into cells

The capacity of fibronectin to bind with α5β1 integrin is now thought to be the most prevalent mechanism for *S. aureus* internalization in endothelial cells and osteoblasts (Khalil et al., 2007).

Fibronectin functions as a bridge between *S. aureus* and osteoblasts. On the one hand, *S. aureus* has two proteins on its surface that may bind to fibronectin: fibronectin-binding proteins A and B (FnBP A/B). Fibronectin, on the other hand, binds to osteoblasts *via* the α5β1 integrin (Hauck and Ohlsen, 2006). This “Fn bridge” enables *S. aureus* to enter osteoblasts *via* internalization. *S. aureus* mutants lacking FnBPs have trouble internalizing in host osteoblasts (Ahmed et al., 2001). Besides, cells missing the integrin β1 subunit do not internalize *S. aureus* in substantial numbers, highlighting the critical function of integrin α5β1 for the uptake process (Fowler et al., 2000). FnBPs bind to Fn *via* a tandem β-zipper structure, which causes a conformational shift in Fn, exposing a cryptic integrin-binding site in Fn, which then interacts with the α5β1 integrin with great affinity (Prystopiuk et al., 2018). Fn creates mechanically strong bridges between FnBPAs on the surface of *S. aureus* and purified integrins that can sustain substantially higher stresses (∼800 pN) than the conventional Fn-α5β1 integrin interaction (∼100 pN). This great mechanical stability lends itself to an invasion model in which binding of Fn to FnBPA *via* a -zipper results in force-induced unfolding and allosteric activation of FnIII domains. This exposes hidden integrin-binding sites, which engage in a robust, high-affinity contact with integrins (Liang et al., 2016). The local recruitment of structural proteins like tensin, vinculin, and zyxin as well as signaling enzymes like Src family protein tyrosine kinases (PTKs) and focal adhesion kinase (FAK) at the site of bacterial attachment is caused by bacteria-induced clustering of integrins. Multiple downstream effectors, including cortactin, are tyrosine phosphorylated as a result of FAK and Src’s joint activity. The effect of cortactin on cytoskeleton rearrangements *via* the Arp2/3 complex or the control of endocytosis by dynamin is most likely how it functions in the internalization of bacteria (Hauck and Ohlsen, 2006).

### Receptors involved in the interaction between *Staphylococcus aureus* and osteoblasts

In addition to binding to the BEM, *S. aureus* can also directly stimulate osteoblasts through its pathogen-associated molecular patterns (PAMPs), resulting in a variety of cellular reactions. PAMPs can bind to pattern recognition receptors (PRR) on osteoblasts, such as toll-like receptors (TLR) and the tumor necrosis factor receptor 1 (TNFR-1). TLRs are a family of 13 mammalian members, each of which mediates an intrinsic signaling pathway and induces specific biological responses against microorganisms (Uematsu and Akira, 2006). TLR-2, TLR-4, and TLR-5 have been observed in osteoblasts. For *S. aureus* infections, TLR2, which can induce the release of antimicrobial peptides (AMPs) (Varoga et al., 2009), is an important relevant receptor involved in this process (Fournier, 2012). TLR2 can recognize ligands with a wide range of structural variations, including proteins, glycopolymers, peptidoglycans, lipoarabinomannan, and lipoproteins/lipopeptides (Oliveira-Nascimento et al., 2012). There is increasing evidence that lipoproteins play an important role in TLR2 activation by staphylococci (Fournier, 2012). TLR-4 and TLR-5 participate in responses against gram-negative bacteria by recognizing lipopolysaccharide and flagellin, respectively, but are not involved in the interaction between *S. aureus* and osteoblasts (Madrazo et al., 2003). According to current research, apart from PRRs like TLRs, *S. aureus* can also interact with epithelial cells and osteoblasts through the extracellular TNFR-1 receptor (Gomez et al., 2004; Claro et al., 2013). TNFR-1 interacts with the protein A of *S. aureus*, which can result in the production of cytokines, osteoblast death, or an imbalanced bone homeostasis (Claro et al., 2011, 2013). Besides, the iron-regulated surface determinant-B (IsdB) of *S. aureus* was involved in invasion, and IsdB most likely interacts with integrins that bind ligands with the RGD motif (Zapotoczna et al., 2013); however, the endocytic pathway has not been discovered.

### Intracellular *Staphylococcus aureus* and persistence into osteoblasts

In bone infection, *S. aureus* internalization in osteoblasts is crucial. Intracellular persistence process depends on the total number of infected cells (Bongiorno et al., 2020). This transformation from acute to chronic bone infection indicates the initiation of persistent infection caused by survival bacteria in cells. *S. aureus* can survive in osteoblasts following internalization due to two factors (Figure 1): vesicle escape and small-colony variant (SCV) formation. The co-localization of fluorescent intracellular *S. aureus* and a lysosomal-associated membrane marker in osteoblasts revealed the trafficking of live bacteria into late endosomal/lysosomal vesicles, implying that *S. aureus* survives inside the vesicles (Jauregui et al., 2013). The survival and proliferation of *S. aureus* within cells were *via* preventing combination of phagosome and lysosome, subversion autophagy, and others. A recent study shows that internalization of *S. aureus* is higher in macrophages than in osteoblasts, but the proportion of *S. aureus* that survives in osteoblasts is higher than that in macrophages (Hamza and Li, 2014). This disparity results from non-professional phagocytes’ incapacity to remove bacteria from vesicles, allowing *S. aureus* to persist in osteoblasts for long time (Hamza and Li, 2014).

At the same time, these surviving bacteria also transform into SCVs. The SCVs are a slow-growing bacterial subpopulation with abnormal colony shape on agar plates and atypical metabolic properties, which are related to the resistance of *S. aureus*, reinfection, and chronic infections. Such SCVs were produced mostly as a result of antibiotic-induced or spontaneous mutations in particular metabolic genes such as har, hemB, ctaA, and thyA (Chen et al., 2021). Approximately 70% of patients who had long-term antibiotic treatment have *S. aureus* SCV infection (Melter and Radojevic, 2010). SCVs have higher intracellular persistence and lower antibiotic susceptibility than wild-type bacteria (Tuchscherr et al., 2016). *In vitro* exposure to various antibiotics has been demonstrated to produce *S. aureus* SCVs from their parental strains (Zhang G. et al., 2018). The membrane potential of the SCV is lowered, which indirectly lowers the bactericidal efficacy of antimicrobial drugs, because transmembrane potential is necessary for the uptake of positively charged AMPs and antibiotics. In addition, the cell membrane is hydrophobic, yet most antibiotics are hydrophilic, making antibiotic entry into the cell problematic and allowing bacteria to evade the activity of most antibiotics. Furthermore, after escaping the original cells and infecting new cells, SCVs quickly revert to the wild-type, extremely toxic, invasive phenotype, which explains why chronic osteomyelitis patients suffer recurring infections.

## Phase I and II treatment strategies

The first and second phases of treatment focus on killing off free bacteria and preventing their adhesion and internalization into cells. Adhesion and internalization are immediate events. However, given the good bactericidal effect of conventional antibiotics on free *S. aureus*, the current research focuses on preventing the adhesion and internalization of bacteria. *S. aureus* adhesion to the ECM is achieved mainly by its cell wall-anchored (CWA) proteins. CWA proteins are essential virulence factors for the survival of *S. aureus* in the commensal state and during invasive infections. CWA proteins have a variety of functions, including adhesion and invasion of host cells and tissues, escape from immune responses, and biofilm formation (Foster et al., 2014). Therefore, CWA proteins have attracted much attention as therapeutic targets. Moreover, some microbiological agents have been shown to inhibit the adhesion of *S. aureus*. Because the bacteria are free in the first phase, traditional antibiotic use is also necessary.

### Immunotherapy

Recombinant CWA proteins were recently used as the specific vaccines against *S. aureus* infection mainly through antibody-mediated protective immunity (Foster et al., 2014). After being injected into the body, the recombinant CWA protein causes the body to produce antibodies that attack specific CWA proteins on *S. aureus*, thereby inhibiting their function. Many specific vaccines have been developed against various CWA proteins, but most have not yet been subjected to clinical testing. Clumping factor A (ClfA), which is produced by most clinical isolates of *S. aureus*, has been regarded as an important vaccine candidate (Scully et al., 2015; Li et al., 2016). It has been shown to provide partial protection against *S. aureus* infections including lethal bloodstream infections and septic arthritis (Josefsson et al., 2001; Scully et al., 2015; Schneewind and Missiakas, 2019). Similarly, the corresponding vaccines that target clumping factor B (ClfB), or *S. aureus* surface protein X (SasX) have all demonstrated certain abilities to reduce infectivity and/or colonization by *S. aureus* (Liu et al., 2015; Lacey et al., 2019).

However, given the myriad of virulence factors produced by the pathogen, the effect of combinations of CWA antigens is superior to that of a single antigen (Delfani et al., 2016), which supports the use of a combination of antigens in vaccines for future clinical trials (Foster et al., 2014). Yang L. et al. (2016) designed the novel chimeric vaccine IsdB_151–277_ClfA_33–213_ (IC), which is based on the immune-dominant areas of the iron surface determinant B (IsdB) and ClfA. This vaccine induced higher protection in an *S. aureus* sepsis model compared with the single components alone and showed broad immune protection against several clinical *S. aureus* isolates. A surface protein vaccine (containing ClfA, fibrinogen binding protein B, serine-aspartate repeat D, and SpA) raised antigen-specific immune responses that protected leukopenic mice against *S. aureus* bloodstream infections (Rauch et al., 2014). Whereas, some active immunization techniques have not yet proven effective in humans. In a Phase III experiment, an IsdB vaccine that was protective in animals failed to protect patients from major infections following cardiothoracic surgery for unknown reasons (Fowler et al., 2013). There are some possible reasons for the negative findings of clinical trials. Preclinical findings with antigens evaluated in clinical trials were probably exaggerated by vaccine producers. Furthermore, because all human *S. aureus* vaccines developed to date have only targeted one antigen, they are unlikely to protect against complex bacterial infections. Finally, new generation adjuvants, which may be essential in boosting antibody formation and guiding the T-cell response toward the proper profile of cytokine release, were not present in the vaccines (Bagnoli et al., 2012).

Other monoclonal antibodies have also shown good antimicrobial effects. Yang Y. et al. (2016) demonstrated that 2H7, a protective monoclonal antibody targeting the conserved domain of *S. aureus* surface protein A (SasA), could recognize wild-type *S. aureus* and promote the opsonophagocytic killing of *S. aureus*. Besides, Tkaczyk et al. (2016) tested a monoclonal antibody combination targeting alpha toxin (AT) and ClfA that neutralized AT-mediated cytotoxicity, blocked fibrinogen binding by ClfA, prevented bacterial agglutination, targeted the bacteria for opsonophagocytic killing, and provided broad isolate coverage in a lethal-bacteremia mode.

### Microbiological agents

Recently, some microbiological agents have shown excellent effects in inhibiting the invasion of *S. aureus*. Bouchard et al. (2013) demonstrated that *Lactobacillus casei* reduced *S. aureus* Newbould 305 and RF122 internalization by 60–80% without modifying cell viability and morphology. An extracellular anti-inflammatory drug, serratiopeptidase, can reduce the invasion and internalization of *S. aureus* to osteoblasts by 75% (Papa et al., 2013; Selan et al., 2017). In an *in vitro* and *in vivo* experiment, Wang X. et al. (2018) found that the plectasin derivatives MP1102/NZ2114 had a good effect on intracellular *S. aureus* clearance. The inhibition of *S. aureus* internalization by microbiological agents likely involves such means: (a) direct effect on *S. aureus*, including coaggregation, as observed for vaginal lactobacilli (Younes et al., 2012); (b) inhibition of *S. aureus* virulence expression, including major virulence regulators (Papa et al., 2013).

### Enhance the antimicrobial defense of the host cells

Some agents could enhance the antimicrobial defense of the host cells by inducing the production of AMPs and decreasing the secretion of inflammatory cytokines. Sodium butyrate has been shown to increase the expression of tracheal antimicrobial peptide (TAP), β-defensin, and inducible nitric oxide synthase (iNOS) mRNAs in bovine mammary epithelial cells (bMECs), as well as the production of nitric oxide (Ochoa-Zarzosa et al., 2009). Short-chain fatty acids (propionic and hexanoic) have similar actions. Propionic and hexanoic reduced bacterial internalization into bMECs, which ranged 27–55 and 39–65%, respectively. And they up-regulated TAP mRNA expression; however, bovine neutrophil β-defensin 5 (BNBD5) mRNA expression was not modified or was down-regulated (Alva-Murillo et al., 2012). Besides, bMECs treated with 17β-Estradiol (E2) (50 pg/mL, 24 h) reduced *S. aureus* internalization (∼50%). E2 also decreased the secretion of TNF-α and IL-1β as well as IL-6 production. Furthermore, E2 also increased the expression of AMPs DEFB1, BNBD5, and psoriasin S100A7 (Medina-Estrada et al., 2016).

## Phase III treatment strategies

After *S. aureus* adheres to the cell surface, it begins to be internalized into the host cell. The *S. aureus* internalization was an active process that was mainly mediated by the FnBPs and integrin α5β1 of host (Sinha et al., 2000; Hauck and Ohlsen, 2006; Alva-Murillo et al., 2014) cells. There are two main results after *S. aureus* invades host cells: (a) virulence factors produced by the bacteria or the inflammatory reaction induced by the bacteria cause most host cells to rapidly lyse and die; and (b) some *S. aureus* bacteria transform from the wild type into a less toxic small colony variant type and live in host cells for a long time (Proctor et al., 2014). At present, more research is aimed at this phase of treatment, and many types of treatments have been demonstrated to have a good bactericidal effect on intracellular *S. aureus*.

### Nanoparticles

The effectiveness of nanoparticles loaded with antibiotics to treat bacterial infections has been studied for many years due to their excellent characteristics including their nano size, surface charge, and large specific surface area. Nanoparticles have contributed to great progress in research on antibacterial biofilms, and they can effectively penetrate thick biofilms and bacterial membranes and disrupt these membranes to kill bacteria (Mu et al., 2016; Mihu et al., 2017). Furthermore, because nanoparticles can effectively penetrate the cell membrane and improve the concentration and bactericidal activity of antibiotics in cells, the field of nanotechnology provides novel approaches for tackling internalized *S. aureus* (Maya et al., 2012; Mu et al., 2016). Given the different types of nanoparticle carriers, the corresponding bactericidal effect is not the same.

Nanoparticles can improve the permeability and accumulation of their payload drug within cells. Increased cellular uptake and the subsequent controlled release of the nanoparticle-adsorbed antibiotics can effectively enhance their antibacterial effects, which makes them more effective for treating intracellular infection (Zhou et al., 2018). Silver nanoparticles (AgNPs) and their combination with antibiotics have demonstrated high extracellular and intracellular bacterial killing abilities and present unique aspects for potential clinical applications (Kang et al., 2019). Maya et al. (2012) developed biocompatible, 200-nm-sized tetracycline-encapsulated O-carboxymethyl chitosan nanoparticles (Tet-O-CMC Nps). These Tet-O-CMC Nps were capable of delivering Tet intracellularly and showed a sixfold increase in antibacterial activity over that of free Tet against intracellular *S. aureus*. To improve the efficiency of delivery and target specificity, Yang Y. et al. (2016) reported a unique intracellular antibiotic delivery nanoparticle that is composed of (a) a mesoporous silica nanoparticle core loaded with gentamicin, (b) an infected microenvironment (bacterial toxin)-responsive lipid bilayer surface shell, and (c) the bacteria-targeting peptide ubiquicidin (UBI29.41), which is immobilized on the lipid bilayer shell surface. BI29.41 showed high sensitivity, specificity, and accuracy for detecting bacterial infection.

Due to their unique biologic performance, nanoparticles armed with antimicrobial agents are used as a potential weapon against *S. aureus* infection and have demonstrated more advantages than the conventional preparations. However, study on nanosystems’ ability to treat *S. aureus* infections is still ongoing, and we must contend with issues like reasonable large-scale production and the premature release of nanoparticles. Besides, there are currently few nanoparticles designed to treat the SCV phenotype of *S. aureus*. New approaches for the production of nanoparticles should be established in order to eradicate the SCV phenotype (Zhou et al., 2018).

### Photochemical internalization

Photochemical internalization (PCI) is a physical-targeting technique in which amphoteric photosensitizers located in endocytic vesicles undergo a series of chemical reactions based on light excitation at a specific wavelength. This in turn causes the vesicle to burst, and the large molecules in the vesicle are released into the cytoplasm. PCI has been shown to facilitate entry into the cytoplasm of most large molecules, including nucleic acids and proteins, and other molecules that cannot easily penetrate cell membranes (Jerjes et al., 2020a,b; Sosic et al., 2020).

Zhang X. et al. (2018) demonstrated for the first time that PCI can effectively enhance the cytosolic release of antibiotics from endocytic vesicles after internalization, thus providing a good clearance effect of intracellular *S. aureus*. First, antibiotics, together with photosensitizers, are internalized through endocytosis or phagocytosis. This process encases the internal antibiotics in vesicles. The photosensitizer is localized in the membrane of the intracellular vesicle, and the drug may be isolated inside the vesicle. Upon illumination, these photosensitizer-bound membranes are disrupted, causing the drugs to be released from the vesicles into the cytoplasm and allowing them to reach their intracellular targets. Thus, with PCI, a lower antibiotic dose can be used for treating (intracellular) staphylococcal infection. Whereas, there are relatively few studies about PCI for treating intracellular *S. aureus* at present, and more research is needed in the future to overcome issues such as PCI toxicity.

### Cell penetrating peptides

CPPs are a family of various peptides, typically comprising 5–30 amino acids, that can pass through tissue and cell membranes (Guidotti et al., 2017). These peptides have been extensively shown to be capable of transporting a wide variety of biologically active conjugates (cargoes) into cells including proteins, peptides, DNAs, siRNAs, and small drugs, and thus they are considered efficient drug delivery vehicles (Mahmood et al., 2016). Cargoes can be conjugated to CPPs either by covalent bonds or by non-covalent complex formation (Guidotti et al., 2017). The mechanism of how CPPs transport cargoes from outside the cell to inside the cell has been extensively studied in recent years, but it is still not completely clear. Nonetheless, these mechanisms of entry can be roughly divided into two categories: energy-independent direct penetration of the plasma membrane and energy-dependent endocytosis; most CPPs and CPP–cargo conjugates enter cells *via* endocytosis (Guidotti et al., 2017). However, precisely determining the transmembrane pathway of a CPP is not that simple. Even for the same CPP, the transmembrane efficiency and pathway may be affected by the concentration, temperature, and cell type (Fretz et al., 2007).

Randhawa et al. (2016) investigated the effects of two CPPs (P3 and P8) in combination with four antibiotics (viz. oxacillin, erythromycin, norfloxacin, and vancomycin) against MRSA strains. They found that this combination of CPPs and antibiotics showed high toxicity against MRSA compared with antibiotics alone. In addition to binding with antibiotics, CCIs can cooperate with some molecules to create an intracellular bactericidal effect. Peptidoglycan hydrolases (PGHs) have good bactericidal activity against both drug-sensitive and -resistant *S. aureus* bacteria. Based on this finding, Rohrig et al. (2020) reported that synergistically active PGH-CPP cocktails reduced both intracellular and drug-resistant *S. aureus.* JDlys is a cell wall hydrolase (also called lysin) derived from staphylococcus phage JD007. The results of an experiment showed that CPP-JDlys can enter keratinocytes and effectively eliminate intracellular MRSA. In further experiments in mice, CPP-JDlys efficiently inhibited the proliferation of MRSA in murine skin and thus shortened the course of wound healing (Wang Z. et al., 2018). However, the clinical effectiveness of CPPs as drug delivery vehicles is hampered by a number of characteristics. The biggest obstacles for CPP-based medications are physiological instability, a lack of selectivity, and low efficacy (Kim et al., 2021).

### Antimicrobial peptides

AMPs, which are small proteins with potent antibacterial, antiviral, and antifungal activity, are widely found in nature. More than 3,100 different AMPs have been described thus far (Lazzaro et al., 2020). AMPs are secreted by cells in the body as the first barrier of defense against microbial invasion. Some of these AMPs are present constitutively while others may be induced in response to infection (Alcayaga-Miranda et al., 2017). AMPs have a positive charge, which attracts them to the generally negatively charged membranes of bacteria; this results in pore formation and membrane perturbation, leakage of cellular components, and cell death (Stallmann et al., 2006; Lazzaro et al., 2020).

Recently, some short synthetic AMPs have demonstrated highly effective intracellular antimicrobial activity. Bormann et al. (2017) reported that short artificial AMPs that contain three arginine residues and one lysine residue might be responsible for the effective cell penetration observed and killing of internalized bacteria without harming the host cells. Cathelicidin LL-37 is amphiphilic in nature and is comprised of hydrophobic and hydrophilic residues aligned on opposite sides of the peptides. Noore et al. (2013) found that LL-37 was more effective in killing extra- and intracellular *S. aureus* than commonly used conventional antibiotics. However, the cell-penetrating mechanism of AMPs is not fully understood and might occur by direct translocation, endocytotic uptake, or the formation of inverted micelles (Bormann et al., 2017). Besides, natural AMPs are difficult to produce because of their low yield and undesirable impurities. Many AMPs have high antibacterial activity, but some of them have undesirable properties that make them unsuitable for clinical use (Biswaro et al., 2018).

### Bacteriophage therapy

In addition to the more advanced treatments studied above, other new approaches have emerged. A phage is a type of bacteria-targeting virus that has extremely high specificity and is easy to apply. A specific phage can only kill the targeted bacteria but does not affect the normal bacteria and body cells (Grunenwald et al., 2018). Phages infect their specific bacterial hosts and during the lytic (or virulent) lifestyle highjack the machinery of the host cell to replicate and ultimately destroy the host, thus simultaneously producing progeny and killing the host. When used as a bactericidal drug, phages have the following characteristics (Nikolich and Filippov, 2020): (a) phages are effective against multi-drug resistant pathogens and can therefore be used in combination with antibiotics, often with synergistic effects; (b) phages are highly specific and usually infect only one bacterium or subgroup, so they have little effect on the normal microflora; (c) a phage is a self-replicating drug because it can replicate on the target bacteria and concentrate precisely where the pathogen cells at the site of infection must be destroyed; (d) since phages coevolve with their hosts, they can adapt to newly emergent resistant strains of the host bacterium; (e) phages are usually weak immunogens; thus, adverse immunologic responses are unlikely; and (f) when the targeted bacteria disappear, the phage disappears and does not remain in the body.

Through genetic engineering, modified phages can be used for targeted drug delivery and new material assembly, thus extending the field of phage therapy and further expanding the medical community’s understanding of phages (Pires et al., 2016). Kim et al. (2012) found that efficient internalization and cytosolic localization of 3D8 VL transbody-displayed phages provides a useful tool for the intracellular delivery of polar macromolecules. This study also proves that phage internalization occurs *via* a physiological endocytotic mechanism through specific cell surface receptors rather than non-specific transcytotic pathways. By fluorescent labeling of the phage and *S. aureus*, the phage was shown to penetrate bovine mammary epithelial cells and remove *S. aureus* from the cells (Zhang et al., 2017). Phages can also enter macrophages by infecting MRSA, killing the intracellular MRSA, and significantly reducing the cytotoxic damage caused by MRSA (Capparelli et al., 2007; Kaur et al., 2014). Previous studies have shown a synergistic effect between bacteriophages and antibiotics or CCP (Gutierrez et al., 2018; Wang Z. et al., 2018). JDlys is a cell wall hydrolase (also called lysin) derived from Staphylococcus phage JD007. CPPTat-JDlys, a fusion of CPPTat to JDlys, was able to effectively eliminate intracellular MRSA bacteria and alleviate the inflammatory response and cell damage caused by MRSA (Wang Z. et al., 2018). Similarly, the combination of lytic protein CF-301 and daptomycin and endolysin MR-10 and minocycline was found to significantly increase survival from bacteremia in mice (Gutierrez et al., 2018). However, up to now, the phage therapy has limitations related to the bacterial resistance to phage (Caflisch et al., 2019), quality and safety requirements, stability of phage preparations, fast phage screening methods, and unsatisfactory regulatory framework (Pires et al., 2020). More research is needed in the future to refine phage therapy.

## Conclusion

*Staphylococcus aureus* internalization can be divided into three different phases, each with its own characteristics. Once *S. aureus* is internalized into cells, it is difficult to treat even with antibiotics. In the first and second phases, immunotherapy and some microbial agents can effectively interfere with bacterial adhesion. In third phase, nanoparticles, photochemical internalization, cell penetrating peptides, antimicrobial peptides, and bacteriophage therapy can effectively eliminate bacteria from cells. However, it is worth noting that most of these treatments are only in the experimental phase at present and have not entered the clinical trial phase. Significant efforts must be made to verify the therapeutic effect and safety of these drugs.

## Author contributions

JL designed the study, collected the data, and wrote the manuscript. QW and FG critically revised the work. LA contributed to the writing. TY contributed to the review of the manuscript. All authors contributed to the article and approved the submitted version.
